# P-586. Weight Gain in Treatment Naïve Patients with HIV Prescribed with Integrase Strand Transfer Inhibitor (INSTI)

**DOI:** 10.1093/ofid/ofae631.784

**Published:** 2025-01-29

**Authors:** Thomas Ludden, Jeremy Thomas, Brianne Vaught, Douglas Meardon

**Affiliations:** Atrium Health, Charlotte, North Carolina; Atrium Health, Charlotte, North Carolina; Atrium Health, Charlotte, North Carolina; Atrium Health, Charlotte, North Carolina

## Abstract

**Background:**

Advances in care for patients with human-immunodeficiency virus (HIV) have progressed significantly over the past few decades. Integrase strand transfer inhibitors (INSTI) are recommended as initial therapy for HIV treatment-naive patients due to virologic efficacy, tolerability, toxicity profile, and ease of dosing. Current guidelines suggest consideration of weight gain as a possible side effect. As with overall US population trends of increased incidence of obesity, this same trend is being seen in the rate of overweight/obese patients with a new diagnosis of HIV. In recent years, there has been increasing concern regarding weight gain specifically associated with antiretroviral therapy (ART). Prior studies have found that individuals who start or switch to a regimen containing certain integrase inhibitors or tenofovir alafenamide (TAF), especially in combination, are more likely to gain weight. Here we examine the treatment of naïve patients with INSTI by BMI classes (underweight/normal, overweight, and obese).

**Methods:**

Baseline BMI and weight were collected for treatment naïve patients with HIV who were prescribed an INSTI. Patients taking medications considered to be beneficial for weight loss were excluded. Weight one year later (+/- 2 months) was collected and paired with the patient's baseline weight. Average weight change was summarized for all patients and patients in underweight/normal, overweight, and obese BMI categories.

**Results:**

Overall, 220 patients who were prescribed INSTI began with an average weight of 83.9 kgs at baseline and increased to 86.8 kgs 1 year later with an average of 2.93 kgs ([95%] CI 1.385 – 4.484), P< 0.001). For the 86 patients with underweight/normal BMI, weight increased by 3.12 kgs ([95%] CI 1.754 – 4.484), P< 0.001). For the 72 patients with overweight BMI, weight increased by 2.30 kgs ([95%] CI 0.076 – 4.515), P=0.043). For the 62 patients with baseline BMI classified as obese, weight increased by 3.42 kgs ([95%] CI -1.17 – 8.016), P=0.141).

**Conclusion:**

Treatment naïve patients with HIV prescribed with INSTI have statistically significant weight gain. These increases were also significant for patients with normal and overweight BMI at baseline but not for patients with a BMI classified as obese.

**Disclosures:**

**All Authors**: No reported disclosures

